# Longitudinal analysis of SARS-CoV-2 IgG antibody durability in Puerto Rico

**DOI:** 10.1038/s41598-024-80465-4

**Published:** 2024-12-28

**Authors:** Zachary J. Madewell, Nathan E. Graff, Velma K. Lopez, Dania M. Rodriguez, Joshua M. Wong, Panagiotis Maniatis, Freddy A. Medina, Jorge L. Muñoz, Melissa Briggs-Hagen, Laura E. Adams, Vanessa Rivera-Amill, Gabriela Paz-Bailey, Chelsea G. Major

**Affiliations:** 1 Division of Vector-Borne Diseases, Centers for Disease Control and Prevention, San Juan, Puerto Rico; 2 Coronavirus and Other Respiratory Viruses Division, Centers for Disease Control and Prevention, Atlanta, Georgia; 3 Eagle Health Analytics, San Antonio, Texas USA; 4 Division of Bacterial Diseases, Centers for Disease Control and Prevention, Atlanta, Georgia; 5 Ponce Health Sciences University/Ponce Research Institute, Ponce, Puerto Rico

**Keywords:** Antibody dynamics, Omicron, Caribbean, COVID-19, Vaccination, Humoral immunity, Epidemiology, Viral infection

## Abstract

Understanding the dynamics of antibody responses following vaccination and SARS-CoV-2 infection is important for informing effective vaccination strategies and other public health interventions. This study investigates SARS-CoV-2 antibody dynamics in a Puerto Rican cohort, analyzing how IgG levels vary by vaccination status and previous infection. We assess waning immunity and the distribution of hybrid immunity with the aim to inform public health strategies and vaccination programs in Puerto Rico and similar settings. We conducted a prospective, longitudinal cohort study to identify SARS-CoV-2 infections and related outcomes in Ponce, Puerto Rico, from June 2020–August 2022. Participants provided self-collected nasal swabs every week and serum every six months for RT-PCR and IgG testing, respectively. IgG reactivity against nucleocapsid (N) antigens, which generally indicate previous infection, and spike (S1) and receptor-binding domain (RBD) antigens, which indicate history of either infection or vaccination, was assessed using the Luminex Corporation xMAP® SARS-CoV-2 Multi-Antigen IgG Assay. Prior infection was defined by positive RT-PCRs, categorized by the predominant circulating SARS-CoV-2 variant at the event time. Demographic information, medical history, and COVID-19 vaccination history were collected through standardized questionnaires. Of 882 participants included in our analysis, 34.0% experienced at least one SARS-CoV-2 infection, with most (78.7%) occurring during the Omicron wave (December 2021 onwards). SARS-CoV-2 antibody prevalence increased over time, reaching 98.4% by the final serum collection, 67.0% attributable to vaccination alone, 1.6% from infection alone, and 31.4% from both. Regardless of prior infection status, RBD and S1 IgG levels gradually declined following two vaccine doses. A third dose boosted these antibody levels and showed a slower decline over time. N-antibody levels peaked during the Omicron surge and waned over time. Vaccination in individuals with prior SARS-CoV-2 infection elicited the highest and most durable antibody responses. N or S1 seropositivity was associated with lower odds of a subsequent positive PCR test during the Omicron period, with N antibodies showing a stronger association. By elucidating the differential decay of RBD and S1 antibodies following vaccination and the complexities of N-antibody response following infection, this study in a Puerto Rican cohort strengthens the foundation for developing targeted interventions and public health strategies.

## Introduction

The ongoing COVID-19 pandemic has spurred extensive research into diagnostic methods for current infections. Reverse transcription polymerase chain reaction (RT-PCR) and antigen tests are vital for detecting current infections, but retrospective identification of SARS-CoV-2 infections is also important for informing public health measures such as vaccine development and for understanding transmission dynamics. Serological assays offer rapid and reliable tools for identifying prior infections by detecting antibodies following SARS-CoV-2 infection^1,2^ These assays commonly target immunoglobulins M (IgM) and G (IgG) antibodies in humans^3^

IgG antibodies are generally detectable for longer periods after infection compared to IgM and potentially play a role in long-term immunity following SARS-CoV-2 infection or vaccination. During the human immune response to SARS-CoV-2, IgG conversion typically occurs around 14 days post-infection, with antibodies remaining detectable for up to at least 15 months^4–7^ Recent studies have revealed variations in immune responses to COVID-19 vaccines, with mRNA vaccines eliciting higher antibody affinity and resulting IgG titers compared to other vaccine types^8–10^ Receiving three or more doses of mRNA vaccines have been shown to provide greater IgG durability than completion of just two vaccine doses^9^ offering strong protection against hospitalization with vaccine effectiveness estimates of 82.5% (95% CI 77.8%–86.2%) after the third vaccine dose and 87.3% (95% CI 75.5%–93.4%) after the fourth dose^11^ Hybrid immunity, resulting from both previous infection and vaccination, has been reported to provide better protection compared to infection or vaccination alone^12^ Serological assays that detect IgG antibodies against multiple SARS-CoV-2 proteins can be valuable in differentiating between previous infection and vaccination, as well as assessing potential differences in resulting immunity duration. However, knowledge gaps remain regarding the longevity of these protective responses, particularly with the emergence of new variants.

Key IgG targets include the nucleoprotein (N) and the spike (S1) glycoprotein’s receptor-binding domain (RBD), both major structural viral proteins of SARS-CoV-2^13–15^ S1 interacts with the human ACE2 receptor, facilitating viral entry, whereas RBD is a specific binding site within S1 targeted by neutralizing antibodies^16^ Understanding the persistence of these antibodies post-infection is essential for evaluating immunity, especially in the context of vaccination. S1 and RBD antibodies may indicate both infection-induced and vaccine-induced immunity. Conversely, N antibodies primarily indicate infection-induced immunity due to the exclusion of N protein in current FDA-approved vaccines which focus on eliciting an immune response that targets S1. In settings with high vaccination rates, N antibody assays can be helpful in distinguishing prior SARS-CoV-2 infections from vaccination-induced responses, especially for mild or asymptomatic infections which often go undetected during their acute phase^17^.

Waning of infection-induced or vaccine-induced antibodies over time is a concern, particularly with the emergence of new SARS-CoV-2 variants^18,19^ Different variants may elicit diverse antibody responses and be variably affected by immunity from prior infections or vaccination. For example, infection with the Omicron variant has been shown to result in a higher anti-N IgG response than other variants, particularly among vaccinated individuals^20^ The Omicron variant showed greater resistance to IgG neutralization generated from early (wild-type) infection or original COVID-19 monovalent vaccines, leading to relatively high infection rates in previously infected and vaccinated individuals^6,21^ In light of these findings, further characterization of immune response durability is necessary. This includes investigating how factors such as age, underlying health conditions, and the combined effect of vaccination and infection history influence IgG persistence and resulting immunity.

This study uses a unique Puerto Rican cohort, providing novel insights into antibody responses within a semi-isolated Caribbean population, distinct from mainland U.S. studies where vaccination rates and variant exposures differed. This is the first longitudinal analysis of its kind in Puerto Rico, investigating SARS-CoV-2 IgG antibody responses (N, S1, RBD) during 2020–2022. The primary outcomes include the levels and durability of IgG antibodies, stratified by vaccination status, history of infection, age, and chronic conditions. We also assess potential waning of antibodies over time, variations in antibody responses based on the presumed SARS-CoV-2 variant (based on sample collection timing), and the sequence of vaccination and infection. In addition, we estimate the prevalence and persistence of hybrid immunity and analyze associations between IgG seroreactivity and subsequent SARS-CoV-2 infections. These analyses aim to enhance our understanding of long-term immunity against SARS-CoV-2, informing public health strategies and vaccination programs in Puerto Rico and similar settings.

## Methods

### Study design and population

Communities Organized to Prevent Arboviruses (COPA) is an ongoing cohort study launched in 2018 in Ponce, Puerto Rico, to assess arbovirus burden and control interventions in a community-based population. COPA is a collaboration involving Ponce Health Sciences University/Ponce Research Institute, the Puerto Rico Vector Control Unit, and the U.S. Centers for Disease Control and Prevention (CDC). Study enrollment and data collection activities are described elsewhere^22,23^.

A COPA sub-study for COVID-19, COCOVID, was implemented in June 2020, enrolling COPA participants and other residents from 15 selected community areas^24,25^ Eligible individuals were ≥ 1 year old, spent four or more nights a week in the selected residence, and had no definite plans to move in the next 12 months. Primary study enrollment occurred from June 2020 to February 2021, with subsequent secondary enrollment until April 2022 limited to household members of active participants. Participants answered questionnaires and provided serum for multi-IgG antibody testing at enrollment and every 6 months until August 2022. They also provided weekly self-collected anterior nasal swabs for SARS-CoV-2 RT-PCR testing until April 2022. Additional anterior nasal swabs for expedited RT-PCR testing were collected by study staff for participants with COVID-like symptoms or close contact with a COVID-19 case during the entire COCOVID study period and until February 2023 for all participants that remained active in the COPA cohort.

### IgG antibody testing

The Luminex xMAP^®^ SARS-CoV-2 Multi-Antigen IgG Assay^26^ was used according to the manufacturer’s instructions to assess seroreactivity in COCOVID serum specimens against three key antigen components: N, S1, and RBD^27^ The multiplex assay uses Luminex MagPlex beads for each IgG antigen target, and specimens were considered positive for N, S1, or RBD when their median fluorescence intensity (MFI) was > 700 call threshold. The MFI is considered a measure of the total degree of saturation in the Luminex test platform and was used as a surrogate marker for the level of antibody titers in our analyses.

### Infection events and status

SARS-CoV-2 infection was defined by positive RT-PCR results from nasal swabs collected weekly throughout the study period (June 2020–August 2022), including up to 6 months after the participants’ final serological test. In our analyses, participant infection status at each serum collection date was assigned based on RT-PCR test results from all previously collected swabs, either as having had one or more previous RT-PCR positive results or all negative previous RT-PCR results. Positive tests separated by ≥ 90 days and accompanied by at least one negative test in between were considered separate illness episodes^28^.

Based on dominant variants circulating in Puerto Rico, infection events that occurred prior to and after serum was collected for IgG testing were categorized by collection date of first positive nasal swab: pre-Delta (June 2020–May 2021), Delta (June–November 2021), and Omicron (December 2021–February 2023)^29,30^; pre-Delta variant circulation periods were not individually defined due to low sample size. For participants with more than one apparent infection event within a 6-month period, the variant circulation period for the most recent infection was assigned.

### Vaccination status, hybrid immunity, and other participant characteristics

Questionnaires answered by participants at enrollment and every 6 months during follow-up included data collection on demographics, health conditions, and COVID-19 vaccination status, including the number of doses, date of each dose, and manufacturer (Pfizer-BioNTech’s BNT162b2, Moderna’s mRNA-1273). Serum specimens were categorized based on the number of COVID-19 vaccine doses the participant had received prior to the collection date: unvaccinated (no doses), 1 dose, 2 doses, and 3 doses.

Consistent with previous research^31^, for the sub-analysis of hybrid immunity, vaccine-induced immunity was defined as history of at least one COVID-19 vaccination confirmed by vaccination cards, infection-induced immunity was defined as anti-N and anti-S1 antibody detection, and hybrid immunity was defined as combined protection from ≥ 1 dose of COVID-19 vaccination, and anti-N and anti-S1 antibody detection.

Sera were also categorized by participant age group at the time of collection (< 20 years, 20–39 years, 40–64 years, ≥ 65 years), and whether they had been previously diagnosed with any chronic health condition by a medical provider. Any chronic health condition included reporting one or more previously diagnosed physical or mental health conditions, and serum specimens from participants with and without specific physical conditions, including hypertension, chronic respiratory disease, diabetes, and high triglycerides, were also compared.

### Sample population

We analyzed blood samples (serum specimens) collected at 6-, 12-, and 18-month follow-up visits from participants in the COCOVID study (December 2020–August 2022). Blood samples from enrollment and the 24-month visit were excluded due to incomplete data on past infections using RT-PCR tests. Weekly anterior nasal swab collections for all participants ended in April 2022, before any participant reached their 24-month visit. However, RT-PCR results from anterior nasal swabs up to the 18-month visit were used to categorize IgG responses. All RT-PCR results throughout the study period, including weekly anterior nasal swabs up to April 2022 and additional swabs collected for symptomatic testing or close contact investigation, were considered to identify infections that occurred after vaccination or a documented prior infection. Blood samples from 6-, 12-, and 18-month visits were included if participants met one of the following criteria: 1) completed at least 80% of RT-PCR tests within the preceding 6 months, or 2) had a positive RT-PCR test result within the preceding 6 months. Due to the low number of participants receiving Johnson & Johnson’s single-dose Ad26.COV2.S vaccine in this cohort, analyses excluded serum specimens from those who only received this vaccine. However, participants who received at least one dose of an mRNA vaccine after an initial Ad26.COV2.S dose were included.

### Statistical analysis

The prevalence of vaccine-induced, infection-induced, or hybrid immunity was estimated for four time periods (Dec 2020–Apr 2021, May–Sep 2021, Oct 2021–Feb 2022, Mar–Aug 2022), stratified by age group. SARS-CoV-2 IgG anti-N, anti-RBD, and anti-S1 responses were assessed by days since last COVID-19 vaccine dose and vaccination status, days since RT-PCR positivity, and sequence of RT-PCR positivity and vaccination for those completing a two-dose primary series. Medians and interquartile ranges (IQR) for the MFI were calculated for each of the three IgG responses by vaccination status, days since vaccination (< 14, 14–27, 28–89, 90–179, ≥ 180), and months since RT-PCR positivity (< 2, 2–4, ≥ 5, no previous positive RT-PCR). All MFI values were log-transformed. For participants without prior RT-PCR positivity and negative anti-N antibodies, we evaluated anti-S1 and anti-RBD seropositivity by days since their last vaccine. Anti-N seropositivity rates were examined in previously infected individuals by vaccination status. Fisher’s exact test was used to compare N seropositivity rates across vaccination groups for overall, Pre-Omicron, and Omicron periods.

Generalized linear mixed-effects regression, with participant ID included as a random effect, was used to evaluate unadjusted and adjusted associations between anti-N, anti-RBD, and anti-S1 IgG responses and time since RT-PCR positivity, time since last vaccine dose, and chronic conditions (see Appendix S1 for additional details). Logistic regression with a random intercept was used to analyze associations between IgG seroreactivity and RT-PCR confirmed infection in subsequent six months during the Omicron predominant period. All available data from participants at each follow-up visit was included in the analysis, regardless of their subsequent participation status. All analyses were done using R software, version 4.3.1 (R Foundation for Statistical Computing, Vienna, Austria).

### Ethics declarations

Local ethics review and approval for the COPA project and COCOVID substudy protocols were obtained through the Ponce Medical School Foundation, Inc’s institutional review board (study #171110-VR). CDC also reviewed COPA and COCOVID protocols and determined the activities were conducted in a manner consistent with applicable federal law and CDC policy (refer to 45 C.F.R. part 46; 21 C.F.R. part 56). The authors declare that all methods were carried out in accordance with relevant guidelines and regulations. The authors declare that informed consent was obtained from all subjects.

## Results

### Study population

Among the 1,030 participants enrolled in the COCOVID study, 789 (76.6%) had qualifying serologic test results available at 6 months, 774 (75.1%) at 12 months, and 567 (55.0%) at 18 months following study enrollment (Table S1). In total, there were 882 unique participants with one or more serologic test results included in our analyses. Median age of these participants at baseline was 37 years (IQR: 18–49), 52.7% were female, and 59.3% had any chronic health condition, of which hypertension (22.4%), respiratory illness (17.2%), and diabetes (11.6%) were most frequently reported (Table 1). By their final serologic test, 86 (9.8%) participants were unvaccinated, 10 (1.1%) had received one dose, 316 (35.8%) had received two vaccine doses, 468 (53.1%) had received three doses, and two (0.2%) had received four doses (Table 1).

**Table 1:** Descriptive characteristics of COCOVID participants, Puerto Rico, 2020–2022.

Characteristic	N = 882 n (%)
**Age in years** (median [IQR])	37 (18–49)
**Age group**	
< 20 years	249 (28.2)
20–39 years	237 (26.9)
40–64 years	300 (34.0)
≥ 65 years	96 (10.9)
**Sex**	
Female	465 (52.7)
Male	417 (47.3)
**Any chronic condition**^**a**^	
Yes	523 (59.3)
No	359 (40.7)
**Follow-up visit**	
6 months	789 (37.0)
12 months	774 (36.3)
18 months	567 (26.6)
**COVID-19 vaccination status recorded on final visit**	
Unvaccinated	86 (9.8)
One dose	10 (1.1)
Two doses	316 (35.8)
Three doses	468 (53.1)
Four doses	2 (0.2)
**Positive SARS-CoV-2 RT-PCR during study**^**b**^	
Any positive RT-PCR	300 (34.0)
Pre-Delta predominant period	28 (3.2)
Delta predominant period	17 (1.9)
Omicron predominant period	263 (29.8)
No positive RT-PCR	582 (66.0)

^a^Any chronic condition included hypertension (N = 198, 22.4%), chronic respiratory illness (N = 152, 17.2%), diabetes (N = 102, 11.6%), thyroid conditions (N = 81, 9.2%), cardiac conditions (N = 80, 9.1%), gastrointestinal problems (N = 75, 8.5%), high triglycerides (N = 72, 8.2%), asthma (N = 60, 6.8%), arthritis (N = 57, 6.5%), neurological disorders (N = 35, 4.0%), anxiety (N = 33, 3.7%), rheumatic conditions (N = 28, 3.2%), depression (N = 27, 3.1%), blood disorder (N = 24, 2.7%), fibromyalgia (N = 24, 2.7%), kidney conditions (N = 24, 2.7%), cancer (N = 23, 2.6%), mental illness (N = 17, 1.9%), physical disabilities (N = 15, 1.7%), liver conditions (N = 14, 1.6%), obesity (N = 14, 1.6%), stroke (N = 9, 1.0%), and other (N = 155, 17.6%).

^b^Pre-Delta predominant period: June 2020–May 2021; Delta predominant period: June–November 2021; Omicron predominant period: December 2021–February 2023.

### SARS-CoV-2 infection

Of the 882 participants with one or more serologic test results, 300 (34.0%) had at least one positive SARS-CoV-2 RT-PCR test during the study period (Table 1), with eight (2.7%) experiencing two separate RT-PCR confirmed infections. By the 18-month serologic test, 20.7% (183/882) had at least one positive RT-PCR test, with 78.7% (n = 144) occurring during the Omicron predominant period (December 2021 onwards). The remaining 119 infections occurred in the 6 months following the final serologic test, all of which occurred during the Omicron predominant period.

### Distribution of vaccine-induced, infection-induced, and hybrid immunity over time

**Fig.1:**
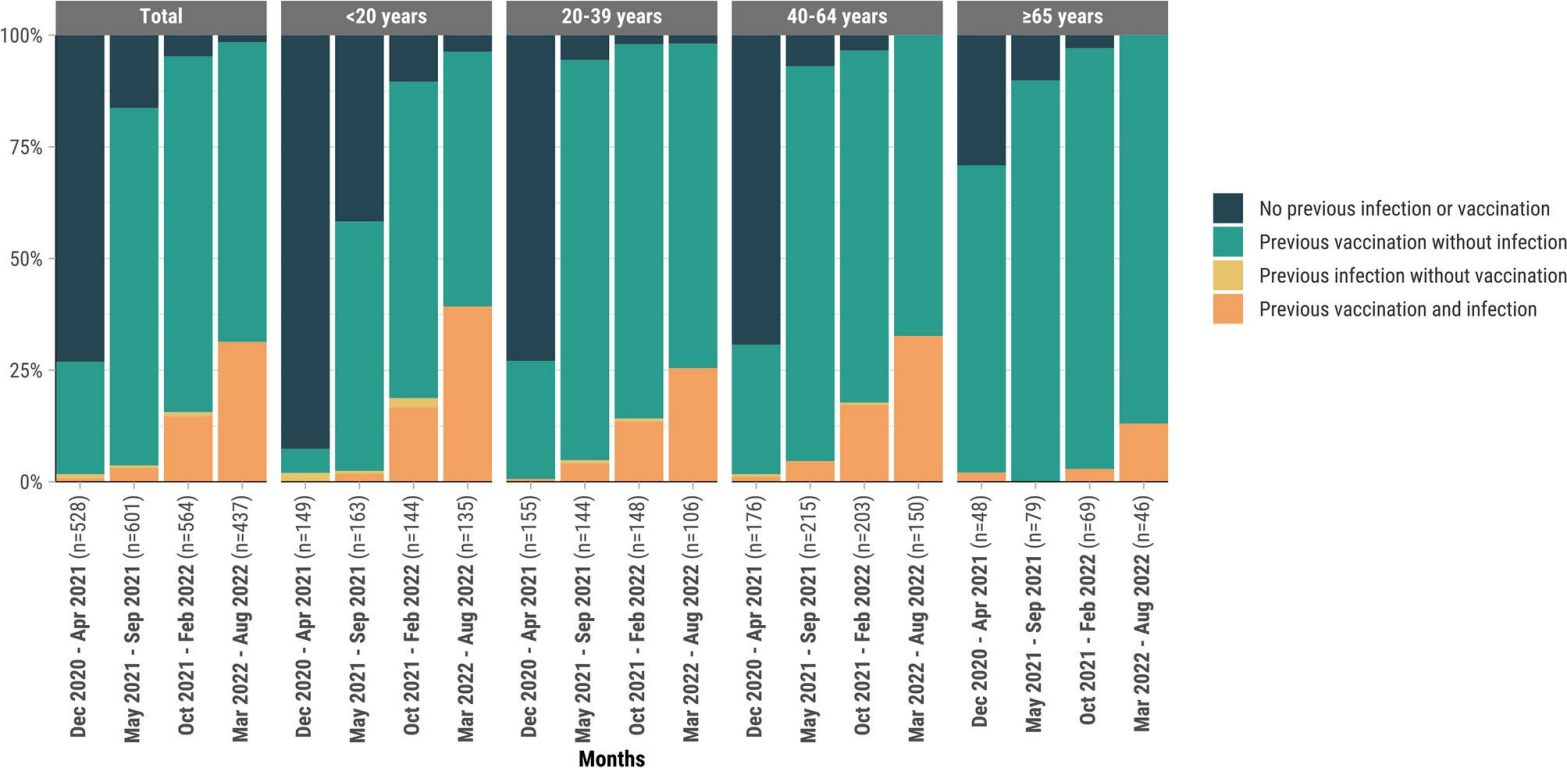
Distribution of vaccine-induced, infection-induced, and hybrid (immunity derived from a combination of vaccination and infection) immunity^a^ against SARS-CoV-2 by age group, Puerto Rico, 2020–2022. ^a^Combined detection of anti-S1 antibodies (produced by both COVID-19–vaccination and SARS-CoV-2 infection) and anti-N antibodies (specific to prior infection), along with vaccination history from vaccination cards. *Vaccine-induced immunity*: Individuals with history of ≥ 1 COVID-19 vaccination dose from vaccination card. *Infection-induced immunity*: Individuals with positive anti-N antibodies and positive anti-S1 antibodies. *Hybrid immunity*: Individuals with self-reported history of ≥ 1 COVID-19 vaccination dose and positive antibodies for both S1 and N antigens.

The prevalence of SARS-CoV-2 antibodies indicative of prior infection or vaccination (both anti-N and anti-S1 positive or anti-N positive only) increased substantially over the study period. In the earliest analysis period (December 2020–April 2021), only 26.9% (95% CI 23.2%, 30.9%) of 528 participants with serological test results had detectable SARS-CoV-2 antibodies. This included individuals with vaccine-induced immunity alone (25.2%), infection-induced immunity alone (0.9%), and hybrid immunity (0.8%) (Fig. 1). By the final period (March–August 2022), 98.4% (95% CI 96.6%, 99.3%) of 437 participants with eligible results had SARS-CoV-2 antibodies, with 67.0% from vaccination alone, 1.6% from infection alone, and 31.4% from both. During this final period, the prevalence of hybrid immunity was highest among participants < 20 years (39.3%, 95% CI 31.1%, 48.1%) and lowest among those ≥ 65 years (13.0%, 95% CI 5.4%, 27.0%).

**Fig.2:**
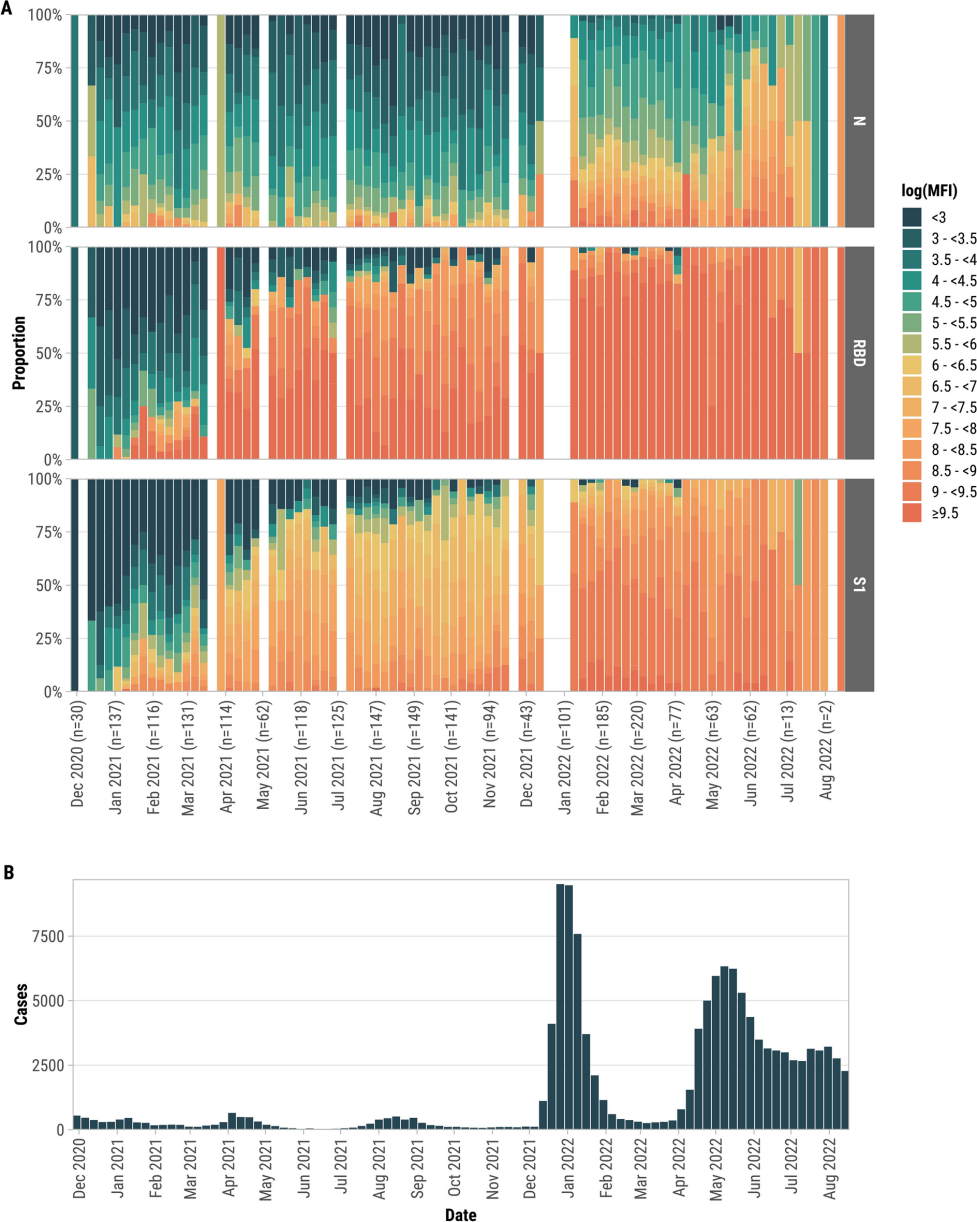
(**A**) Weekly distribution of SARS-CoV-2 N, RBD, and S1 antibody responses during the serosurvey period, represented as proportions of log-transformed median fluorescence intensity (log[MFI]) levels (e.g., < 3, 3–3.5, 3.5–4, etc.). The x-axis labels indicate months, with the number of participants tested each month shown in parentheses. The color gradient ranges from green (lower antibody levels) to red (higher antibody levels, e.g., log(MFI) ≥ 9.5), reflecting the intensity of the immune response over time. (**B**) Weekly COVID-19 case counts (confirmed and probable) reported to the Puerto Rico Department of Public Health for the Ponce Health Region, 2020–2022. These data are not part of the study cohort but are included to provide contextual information on regional COVID-19 trends. Departamento de Salud PR. COVID-19 en cifras en Puerto Rico. 2024 [cited 2024 May 17]; Available from: https://www.salud.pr.gov/estadisticas_v2.

The widespread availability of COVID-19 vaccines in Puerto Rico (April–May 2021) coincided with an increase in RBD and S1 antibody titers (Fig. 2A), as observed through the proportion of higher log(MFI) levels over time. Among participants previously negative for RT-PCR and N antibodies, all who had a serum sample collected within 7–13 days post-second vaccine dose seroconverted for both S1 and RBD antibodies. However, by 90–179 days after the second dose, S1 detection dropped to 75.6%, while RBD remained detectable in 99.1% (Figures S1, S2). A third vaccine dose resulted in 100% detection of both RBD and S1 antibodies at 90–179 days post-vaccination.

N antibody levels increased dramatically during a surge of COVID-19 cases in Puerto Rico following the introduction of the Omicron variant at the end of 2021 (Fig. 2A). This pattern aligns with Fig. 2B, which shows a concurrent increase in regional COVID-19 cases during the same period. Among participants tested during this period, 28.3% (196/692) showed N IgG seroconversion, of whom 75.5% (148/196) had a previous positive RT-PCR test. Among participants who seroconverted for N antibodies, median log(MFI) for N antibodies was higher in participants with a previous positive RT-PCR (8.20, IQR 7.52, 8.70) than those with negative RT-PCRs (7.60, IQR 7.24, 8.61) regardless of vaccination status (*p* = 0.022) (data not tabulated).

### IgG responses and waning by vaccination status

**Fig. 3:**
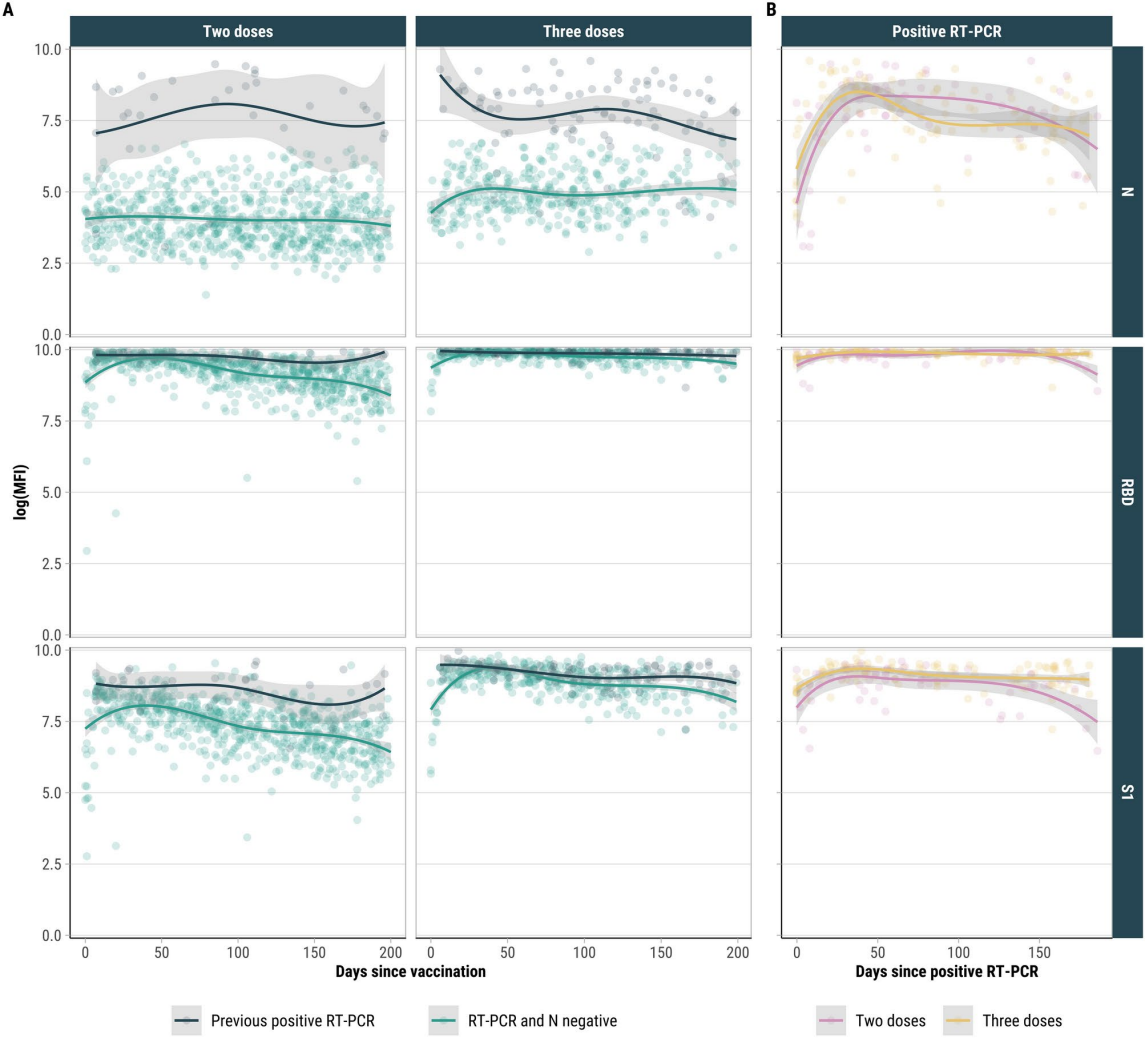
SARS-CoV-2 IgG antibody responses by days since last vaccine stratified by RT-PCR positivity in previous six months vs. no previous RT-PCR positivity and N negative (**A**) and antibody responses by days since last positive RT-PCR and vaccination status (**B**), Puerto Rico, 2020–2022. The lines are loess smoothing lines and shaded bands are 95% CIs.

Analysis of all serum samples suggests that RBD and S1 antibody responses were more commonly detected and persisted longer than N antibody responses across all vaccination groups (Figs. 3, S3). For individuals who received two doses, the overall median log(MFI) at any time post-vaccination was 9.42 (IQR 8.93, 9.70) for RBD, 7.55 (IQR 6.86, 8.17) for S1, and 4.05 (IQR 3.37, 4.87) for N (Figure S3). RBD and S1 antibodies were highly correlated regardless of vaccine dosage (*r* ≥ 0.83) (Figure S4).

In participants who received two vaccine doses without a prior positive RT-PCR, both RBD and S1 levels gradually waned over time (Figs. 3, S3). The median log(MFI) for RBD and S1 decreased from 9.73 (IQR 9.64, 9.83) and 8.09 (IQR 7.73, 8.38) at 14–27 days post-vaccination to 8.65 (IQR 8.24, 9.12) and 6.64 (IQR 6.16, 7.26) at ≥ 180 days, respectively. Younger participants (< 20 years) had higher antibody levels than those aged ≥ 65 at later time points (> 180 days post-vaccination), with a median log(MFI) for S1 of 7.51 versus 6.35 (*p* = 0.020), and for RBD, 9.22 versus 8.48 (*p* = 0.004) (Figure S5, Table S3).

Almost all antibody tests for participants who received a third vaccine dose were conducted during the Omicron predominant period. Among all serum samples collected from participants with a third dose, both RBD and S1 antibody levels peaked at 14–27 days post-vaccination (median log[MFI]: 9.90, IQR 9.85, 9.94, and 9.22, IQR 9.05, 9.47, respectively), declining thereafter to 9.74 (IQR 9.48, 9.90) and 8.79 (IQR 8.33, 9.08) ≥ 180 days post-vaccination, respectively (Figure S3). Adjusting for age group, sex, chronic conditions, and previous infection, log(MFI) for RBD and S1 decreased by -0.002 (95% CI: -0.004, -0.001) and -0.009 (95% CI: -0.012, -0.005), respectively, for each month post-third dose (Figure S6). Statistical comparisons of antibody decay rates confirmed that RBD responses decline more slowly over time compared to both S1 (*Z* = -3.33, *p* < 0.001) and N antibodies (*Z* = 4.45, *p* < 0.001). The decline rates of S1 and N antibodies were not significantly different (*Z* = 1.67, *p* = 0.095). RBD and S1 responses were higher for participants who received a third dose than those who with second doses regardless of age group or time since vaccination (Figure S5).

**Fig. 4:**
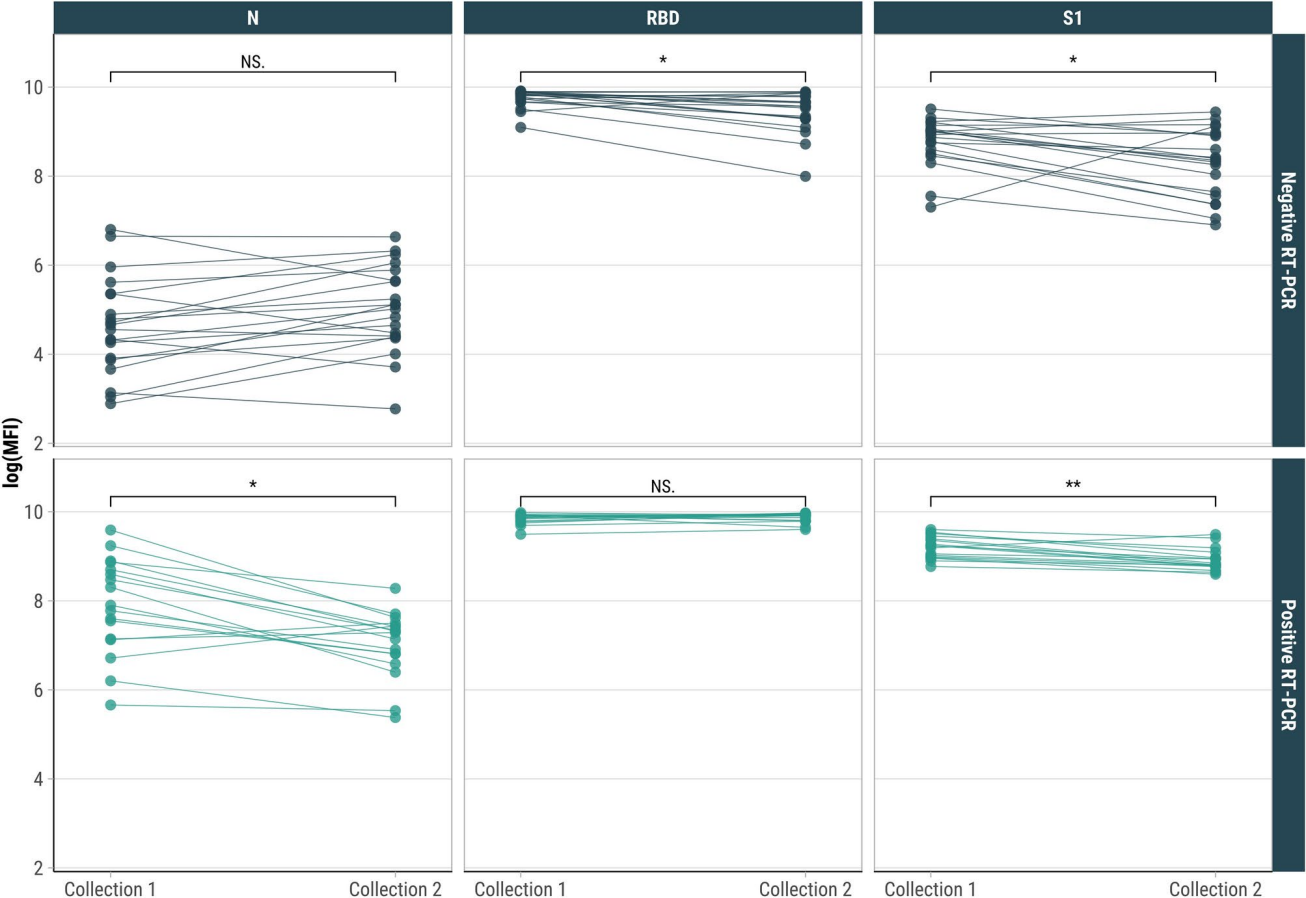
Paired SARS-CoV-2 IgG antibody responses for individuals at two time points six months apart among people who had received three vaccine doses by prior RT-PCR positivity, Puerto Rico, 2020–2022. *P < 0.05, **P < 0.01. NS: not significant.

To directly assess the rate of antibody decline, we analyzed changes in IgG levels between paired serum samples collected at different time points after the second and third vaccine doses in participants with and without prior PCR-confirmed infection. For individuals with a second dose and without a previous positive RT-PCR, RBD and S1 levels fell by 8.8% (from 9.73 to 8.78) and 20.9% (from 8.44 to 6.68), respectively, after six months (Figure S7). For those with a third dose and without a previous positive RT-PCR, RBD and S1 levels fell by 2.3% (from 9.82 to 9.59) and 6.2% (from 8.97 to 8.41), respectively, after six months (Fig. 4). For those with a third dose and a previous positive RT-PCR, S1 levels decreased by 4.1% (from 9.21 to 8.83), while RBD levels remained stable at 9.90 after six months. There were no participants with paired SARS-CoV-2 IgG antibody responses at two time points six months apart with a second dose and a previous positive RT-PCR. N-specific antibodies for sample pairs demonstrate N levels fell by 6.9% (from 7.84 to 7.30) for those with a positive RT-PCR.

### Nucleocapsid responses and RT-PCR positivity

Among individuals with a previous positive RT-PCR (80.8% N seropositive), N antibody levels were higher (median log[MFI]: 7.89, IQR 6.90, 8.56) compared to those without a positive RT-PCR (median log[MFI]: 4.34, IQR 3.60, 5.13) (Figs. 3, S3). N antibody levels peaked within two months of a positive test (median log[MFI]: 8.27, IQR 7.09, 8.69) and gradually declined thereafter, reaching a median log(MFI) of 7.24 (IQR 6.43, 8.30) at five months after testing positive. Among individuals without a history of previous infection, N-seropositivity following an infection was 83.7% and 74.6% for those who received two and three doses, respectively, not significantly different from unvaccinated individuals (77.8%) (*p* = 0.498) (Table 2).

**Table 2:** Anti-N seropositivity rates among participants who tested positive for SARS-CoV-2 by RT-PCR at least 14 days after vaccination and were previously seronegative and RT-PCR negative, Puerto Rico, 2020–2022.

		All		Pre-Omicron		Omicron
Vaccination status	Participants N	Anti-N seropositivity % (95% CI)	Participants N	Anti-N seropositivity % (95% CI)	Participants N	Anti-N seropositivity % (95% CI)
Unvaccinated	18	77.8 (51.9, 92.6)	16	81.2 (53.7, 95.0)	2	50.0 (9.5, 90.5)
Two doses	49	83.7 (69.8, 92.2)	13	69.2 (38.9, 89.6)	36	88.9 (73.0, 96.4)
Three doses	67	74.6 (62.3, 84.1)	–	–	67	74.6 (62.3, 84.1)
Fisher’s exact *p*-value		0.498		0.448		0.154

Fisher’s exact test was used to compare N seropositivity rates across vaccination groups for all, Pre-Omicron, and Omicron periods.

Both vaccination and previous RT-PCR positivity provided a stronger boost to RBD and S1 antibody responses compared to either factor alone (Figs. 3, S3). The order of vaccination and RT-PCR positivity did not significantly impact antibody responses among participants who received two doses (Figure S8).

### IgG seroreactivity and subsequent RT-PCR positivity

Our analysis revealed that individuals with detectable antibodies against either N or S1 were less likely to experience a positive RT-PCR test in the following six months during the Omicron-dominant period. Specifically, N and S1 seropositivity were associated with 90.3% (95% CI 78.8%, 95.6%) and 58.3% (95% CI 12.1%, 80.2%) lower odds, respectively, of a subsequent positive test, after adjusting for age group, sex, and chronic conditions (Table 3). Participants with a higher N antibody level (log(MFI) ≥ 8.55) had an even greater reduction in odds (96.3%, 95% CI 73.0%, 99.5%) compared to those who were N seronegative. These findings suggest that SARS-CoV-2 infection, as indicated by N seropositivity, provided substantial protection against reinfection during the Omicron period, while vaccination, as indicated by S1 seropositivity, also offered significant, though comparatively lower, protection.

**Table 3:** Associations between SARS-CoV-2 seroreactivity and subsequent RT-PCR positivity within six months during the Omicron predominant period, Puerto Rico, 2020–2022.

	**Positive RT-PCR in 6 months after serology** N = 129^a^ n (%)	**No positive RT-PCR in 6 months after serology** N = 625^a^ n (%)	**Odds Ratio** **(95%CI)**	**P-value**	**Adjusted**^b^** Odds Ratio** **(95% CI)**	**P-value**
**S1: seroreactivity**				0.003		0.022
Seropositive	116 (89.9)	602 (96.3)	0.341 (0.168, 0.692)		0.417 (0.198, 0.879)	
Seronegative	13 (10.1)	23 (3.7)	*Reference*		*Reference*	
**S1: log(MFI)**				0.005		0.089
≥ 8.56	77 (59.7)	448 (71.7)	0.304 (0.148, 0.626)		0.407 (0.190, 0.870)	
7.56–8.55	32 (24.8)	116 (18.6)	0.488 (0.223, 1.070)		0.487 (0.214, 1.106)	
6.56–7.55	7 (5.4)	38 (6.1)	0.326 (0.114, 0.936)		0.292 (0.098, 0.873)	
Seronegative	13 (10.1)	23 (3.7)	*Reference*		*Reference*	
**N: seroreactivity**				< 0.001		< 0.001
Seropositive	7 (5.4)	218 (34.9)	0.107 (0.049, 0.234)		0.097 (0.044, 0.212)	
Seronegative	122 (94.6)	407 (65.1)	*Reference*		*Reference*	
**N: log(MFI)**				< 0.001		< 0.001
≥ 8.56	1 (0.8)	184 (29.4)	0.042 (0.006, 0.307)		0.037 (0.005, 0.270)	
7.56–8.55	3 (2.3)	155 (24.8)	0.133 (0.041, 0.431)		0.124 (0.038, 0.403)	
6.56–7.55	3 (2.3)	148 (23.7)	0.156 (0.048, 0.507)		0.140 (0.043, 0.458)	
Seronegative	122 (94.6)	138 (22.1)	*Reference*		*Reference*	

*MFI* median fluorescence intensity.

Almost all (98%) antibody tests were seropositive for RBD, therefore RBD was excluded from this analysis.

^a^The denominator is the number of serological tests performed.

^b^Adjusted for age group, sex, and any chronic condition using mixed-effects logistic regression. All models included participant as a random effect. Associations with S1 were also adjusted for N seroreactivity; associations with N were adjusted for S1 seroreactivity.

### Chronic conditions and IgG responses

Among participants who received two doses, diabetes was associated with lower RBD (β: -0.022, 95% CI -0.039, -0.006) and S1 (β: -0.029, 95% CI -0.056, -0.002) responses compared to those without diabetes, after adjusting for age group, sex, and previous RT-PCR positivity (Figure S9). Similarly, for participants who received a third dose, high triglycerides were associated with lower RBD (β: -0.009, 95% CI -0.016, -0.003) and S1 (β: -0.024, 95% CI -0.040, -0.007) responses compared to those with normal triglyceride levels. No significant associations were found between other chronic conditions and antibody responses.

## Discussion

This study provides novel real-world data on SARS-CoV-2 antibody dynamics in a semi-isolated Caribbean island population throughout the COVID-19 pandemic. This unique setting allows for a more controlled analysis of antibody response compared to geographically dispersed populations. The dataset offers insights into antibody dynamics within a population with distinct vaccination and infection histories compared to mainland populations. The high vaccination rates in Puerto Rico as the initial antigenic exposure to COVID-19, followed by infection with the Omicron variant, provide a unique population to study immunity and waning dynamics, in contrast to many U.S. populations who experienced earlier variants first and subsequent vaccination. We observed a substantial rise in seropositivity (from 26.9 to 98.4%) over the study period, including an increase in hybrid immunity from 0.8 to 31.4%. Studies have shown that hybrid immunity offers several advantages, including enhanced protection against severe disease and death, slower waning of immunity, reduced risk of reinfection, promotion of B cell diversity, and facilitation of T cell activation during subsequent vaccination^32^ However, the impact of this increase on population immunity remains unclear due to the emergence and potential immune escape of SARS-CoV-2 variants. These findings emphasize the importance of ongoing surveillance and tailored vaccination strategies to effectively manage the COVID-19 pandemic.

RBD and S1 antibody levels gradually declined over time following initial vaccine doses, reflecting the natural decay of vaccine-induced humoral immunity. S1 levels waned faster than RBD levels, consistent with previous research^33^ This difference might be due to the inherent structural stability of the RBD region^34^, potentially contributing to slower degradation and potentially longer-lasting protection against viral entry. However, limitations of the assay used warrant further investigation with assays optimized for S1 and RBD detection to definitively assess their relative decay rates. As expected, booster doses substantially increased RBD and S1 levels compared to the primary series, peaking 14–27 days post-booster dose, indicating a rapid and robust immune response.

Recent SARS-CoV-2 infections boosted N, RBD, and S1 antibody levels, peaking within two months and gradually declining after six months, suggesting natural humoral immunity decay. Although infection may provide protective benefits through antibody boosts, vaccination remains the safest strategy for building immunity against COVID-19, considering the risk of severe illness during infection.

One-quarter of participants with N seroconversion during the Omicron-predominant period lacked a positive RT-PCR result. This could be due to asymptomatic or mild infections, transient viral RNA levels below the detection threshold, or infections that were too recent for RT-PCR detection. Alternatively, they could have had an acute infection that was not yet RT-PCR detectable. Supporting this hypothesis, a previous study reported declining RT-PCR sensitivity for detecting asymptomatic cases over time, with peak sensitivity occurring within the first few days after infection^35^ Conversely, some participants with positive RT-PCRs had negative N tests, possibly reflecting early infection before significant antibody development. Alternatively, some individuals with mild or asymptomatic infections might not generate a strong immune response. As a result, their antibody production, including N antibodies, may be minimal or undetectable^36^ These findings highlight the limitations of single testing methods and the importance of considering viral load and timing of testing when interpreting results. They also suggest the potential value of comprehensive testing approaches that combine multiple methods for more accurate diagnosis.

Consistent with previous research^37^, 84% of vaccinated individuals with their first SARS-CoV-2 infection were N-seropositive. In contrast to pre-Omicron studies^17^, we observed no significant differences in N antibody detection between vaccinated and unvaccinated individuals with recent infection. Omicron’s mutations in RBD may enhance breakthrough infections by evading vaccine-induced antibody neutralization^38–40^, potentially leading to increased reliance on and strengthening of the immune system’s N antibody response. Additionally, Omicron has a higher replication rate in the nasopharynx, saliva, and upper respiratory tract compared to ancestral variants^41,42^ This may lead to increased shedding of viral particles, including the N protein, which the immune system can detect and generate antibodies against.

Younger individuals (< 20 years) had higher S1 and RBD levels than adults over 65 years beyond 5 months post-vaccination, consistent with other studies^43,44^ Differences associated with advancing age may be attributed to thymic involution and reduced B cell generation, differences in inflammatory response or cytokine production, and underlying comorbidities or immune modulatory medications^45^ Among the explored comorbidities, diabetes and high triglycerides were associated with lower IgG responses. The inverse association with high triglyceride concentrations could be due to potential interference from triglycerides with the assay used. Although an interfering substances study was not performed for this study, it is a consideration for future investigations. For diabetes specifically, potential mechanisms include a reduced number of circulating helper T cells and impaired antigen presentation, as reported by Soetedjo et al.^46^ Further research is needed to understand the specific immunological mechanisms by which certain comorbidities compromise vaccine-induced humoral immunity. These findings emphasize the need for tailored vaccination strategies, potentially involving adjuvants to enhance immune responses in older adults and considering earlier booster doses for vulnerable populations.

This study included persons vaccinated with monovalent mRNA vaccines, initially designed against the wild-type SARS-CoV-2. While highly effective in preventing severe illness^11,47^, these vaccines faced declining effectiveness as newer variants emerged^48,49^ Over three-quarters of infections in our study occurred during the Omicron wave. Omicron sub-lineages from BA.1 to BA.5 and subsequent variants such as XBB.1.5 and JN.1 demonstrated increased transmissibility and immune escape due to S1 protein mutations, rendering the monovalent vaccines less protective^50^ In response, bivalent mRNA boosters targeting both the original strain and Omicron subvariants (BA.1 or BA.4–5) were developed for use in late 2022 and demonstrated increased effectiveness against severe outcomes with Omicron infection compared to monovalent vaccines in both laboratory and real-world studies^51–53^ COVID-19 vaccine formulations were subsequently updated for 2023–2024 to a new monovalent vaccine, designed to target the Omicron XBB.1.5 sub-lineage^54^ The earlier bivalent and monovalent formulations are no longer authorized for use in the U.S.

Despite waning antibody levels and variant emergence, vaccines remain an important way to prevent severe illness, hospitalization, mechanical ventilation, and death associated with COVID-19^11,53^ Additionally, effective treatments are available, further contributing to the reduction of severe outcomes. This protects individual health and reduces the economic burden on healthcare systems. Treating severe COVID-19 cases is costly, with inpatient stays averaging $11,275 as of March 2022^55^ Widespread vaccination reduces these high-cost hospitalizations, offering clear economic benefits.

This study was subject to several limitations. First, our analysis relied on specific antibody assays measuring IgG levels of N and S1 proteins. However, other relevant immune responses, such as B cell and T cell-mediated immunity, were not assessed. Second, this study focused on antibody levels, not directly assessing their functional effectiveness against variants. Changes in levels may not always translate to altered protection against clinical outcomes. Our findings showed an association between higher anti-N IgG levels and a decreased likelihood of a subsequent positive RT-PCR test. The relationship between antibody levels and disease severity can be complex and may vary depending on factors such as the specific variant, the type of antibodies measured (IgG versus neutralizing antibodies), and individual immune response. However, our findings align with studies suggesting that higher IgG levels may be associated with a lower risk of developing symptomatic COVID-19 infection^56,57^.

Our study describes IgG responses following vaccination and infection among a cohort in Puerto Rico. Understanding the differential decay of RBD and S1 antibodies, the impact of comorbidities, and the nuances of N response following infection further informs targeted interventions and public health strategies. Some observed N and S1 serum reactivity might reflect undetected SARS-CoV-2 exposure, underscoring the value of serological testing in capturing broader population immunity compared to RT-PCR testing, which is only detectable for a short time and might miss asymptomatic or mild cases. Optimal testing to understand viral pathogenesis requires serial determinations of both viral antigens and antibody responses.

## Supplementary Information

Supplementary Information.

## Data Availability

The datasets generated and analyzed during the current study are not publicly available due to privacy and restrictions but are available from the corresponding author (ock0@cdc.gov) upon reasonable request.
